# Association of body mass index with disease severity and prognosis in patients with non-cystic fibrosis bronchiectasis

**DOI:** 10.1590/1414-431X20154135

**Published:** 2015-07-10

**Authors:** Q. Qi, T. Li, J.C. Li, Y. Li

**Affiliations:** 1 Department of Respirology, Qilu Hospital of Shandong University, Jinan, Shandong Province, China; 2 Neurosurgical Intensive Care Unit, the First Affiliated Hospital, Sun Yat-sen University, Guangzhou, Guangdong Province, China

**Keywords:** Bronchiectasis, Body mass index, Prognosis, Survival, Underweight

## Abstract

The objective of this observational, multicenter study was to evaluate the association of body mass index (BMI) with disease severity and prognosis in patients with non-cystic fibrosis bronchiectasis. A total of 339 patients (197 females, 142 males) diagnosed with non-cystic fibrosis bronchiectasis by high-resolution computed tomography were classified into four groups: underweight (BMI<18.5 kg/m^2^), normal weight (18.5≤BMI<25.0 kg/m^2^), overweight (25.0≤BMI<30.0 kg/m^2^), and obese (BMI≥30.0 kg/m^2^). Clinical variables expressing disease severity were recorded, and acute exacerbations, hospitalizations, and survival rates were estimated during the follow-up period. The mean BMI was 21.90 kg/m^2^. The underweight group comprised 28.61% of all patients. BMI was negatively correlated with acute exacerbations, C-reactive protein, erythrocyte sedimentation rate, radiographic extent of bronchiectasis, and chronic colonization by *P. aeruginosa* and positively correlated with pulmonary function indices. BMI was a significant predictor of hospitalization risk independent of relevant covariates. The 1-, 2-, 3-, and 4-year cumulative survival rates were 94%, 86%, 81%, and 73%, respectively. Survival rates decreased with decreasing BMI (χ^2^=35.16, P<0.001). The arterial carbon dioxide partial pressure, inspiratory capacity, age, BMI, and predicted percentage of forced expiratory volume in 1 s independently predicted survival in the Cox proportional hazard model. In conclusion, an underweight status was highly prevalent among patients with non-cystic fibrosis bronchiectasis. Patients with a lower BMI were prone to developing more acute exacerbations, worse pulmonary function, amplified systemic inflammation, and chronic colonization by *P. aeruginosa*. BMI was a major determinant of hospitalization and death risks. BMI should be considered in the routine assessment of patients with non-cystic fibrosis bronchiectasis.

## Introduction

Bronchiectasis is an abnormal, permanent dilatation of the bronchi and bronchioles caused by repeated cycles of airway infection and inflammation (1). Bronchiectasis is usually divided into non-cystic fibrosis (non-CF) bronchiectasis, which affects a heterogeneous population and has various etiologies, and bronchiectasis due to cystic fibrosis (CF). CF is an autosomal recessive genetic disorder that not only affects the lungs, but also damages the pancreas, intestines, liver, sweat glands, and vas deferens. CF is rare in Asian races (2) and is considered to be a disease predominantly of Caucasian origin (3). Therefore, the present study only focused on patients with non-CF bronchiectasis.

Non-CF bronchiectasis is associated with chronic cough and expectoration, frequent respiratory infections, lung dysfunction, and advanced dyspnea. These symptoms impose a significant burden on patients, resulting in worsening of quality of life and premature mortality (4). Extrapulmonary manifestations of non-CF bronchiectasis include muscle dysfunction, decreased exercise capacity, fatigue, and a deteriorating health status (5). Clinically, some patients with non-CF bronchiectasis exhibit weight loss and nutritional depletion. A cross-sectional study of 93 patients with bronchiectasis found that 14% of patients presented with malnutrition as defined by a body mass index (BMI) of <18.5 kg/m^2^ (6). Another study found that the prevalence of malnutrition (defined as a BMI of <20 kg/m^2^) was nearly 30% among patients with bronchiectasis (7). A poor nutritional status was directly related to decreasing pulmonary function, and this link was a proposed predictive factor of morbidity and mortality in patients with chronic respiratory diseases (8). Based on our literature review, whether malnutrition accompanies non-CF bronchiectasis or is an important extrapulmonary feature of non-CF bronchiectasis remains unclear.

Measurement of BMI is a simple method for screening malnutrition. BMI has served as an independent prognostic factor for chronic obstructive pulmonary disease (COPD), with a clear association between a low BMI and increased mortality (9). In 2004, one study described a clear association between a low BMI and increased mortality in patients with end-stage respiratory disease (including 33 patients with bronchiectasis) (7). Likewise, a Turkish study suggested that a high BMI was beneficial for survival in patients with bronchiectasis (10). Thus, in addition to the conventional treatment strategies for non-CF bronchiectasis, attention to the nutritional status may promote more favorable outcomes. However, there are no data regarding the association of BMI with disease severity and prognosis in patients with non-CF bronchiectasis in Asia.

In this study, we evaluated the relationships between BMI and clinical variables of disease severity in patients with non-CF bronchiectasis and explored the predictive factors for the risks of hospitalization and mortality in these patients.

## Material and Methods

### Patients

Four general hospitals (Qilu Hospital of Shandong University, Chest Hospital of Shandong Province, the Second Affiliated Hospital of Shandong University of Traditional Chinese Medicine, and Binzhou People’s Hospital of Shandong Province) in China participated in this observational, multicenter cohort study. Inpatients and outpatients were consecutively recruited from 1 January 2010 to 31 December 2013. Bronchiectasis was diagnosed by high-resolution computed tomography scans of the chest. The presence of bronchiectasis on high-resolution computed tomography images was based on criteria published by McShane et al. (1), including the following: the internal diameter of the bronchus was larger than that of its accompanying vessel (signet ring sign), the bronchus did not taper as it traveled to the periphery of the lung, or the bronchus terminated in a cyst. The underlying etiology of bronchiectasis was determined after performing the tests recommended in the British Thoracic Society guideline for non-CF bronchiectasis (11). Patients were excluded if they had a diagnosis of asthma, COPD, traction bronchiectasis due to lung fibrosis, or malignant tumors. In total, 339 Chinese patients were enrolled in the study and were followed until 1 April 2014. This observational study was approved by the Ethics Committee of Qilu Hospital of Shandong University, and all patients gave informed consent.

### Basic data

The following basic data were recorded for each patient: age, gender, body weight, body height, and smoking history. Total symptom duration in years was calculated from the date of symptom onset to the date of recruitment into this study. BMI was calculated by dividing weight in kilograms by the square of height in meters. Patients were categorized into four groups according to the World Health Organization expert consultation on BMI criteria for Asian populations: underweight (BMI<18.5 kg/m^2^), normal weight (18.5≤BMI<25.0 kg/m^2^), overweight (25.0≤BMI<30.0 kg/m^2^), and obese (BMI≥30.0 kg/m^2^) (12).

### Variables of disease severity

#### Pulmonary function

The pulmonary function indices measured in this study were the forced vital capacity (FVC), forced expiratory volume in 1 s (FEV_1_), FEV_1_/FVC, predicted percentage of FVC, predicted percentage of FEV_1_, and inspiratory capacity using a MasterScreen spirometer (Jaeger, Germany). The ventilatory function of patients with non-CF bronchiectasis was classified into one of four categories according to the American Thoracic Society COPD guidelines: normal ventilatory function, obstructive ventilatory dysfunction, restrictive ventilatory dysfunction, or mixed ventilatory dysfunction (13).

#### Arterial blood gas analyses

Arterial blood gas analyses were performed at rest and in room air with a blood gas analyzer (Radiometer Medical ApS, Denmark). Arterial oxygen tension (PaO_2_), arterial carbon dioxide partial pressure (PaCO_2_), and arterial oxygen saturation were measured while the patients breathed room air. According to the British Thoracic Society guideline for noninvasive ventilation in acute respiratory failure, respiratory failure was defined as a PaO_2_ of <8.0 kPa (60 mmHg), with or without a PaCO_2_ of >6.7 kPa (50 mmHg) by arterial blood gas analyses while breathing air at sea level (14).

#### Acute exacerbations

According to the British Thoracic Society guideline for non-CF bronchiectasis, an acute exacerbation of bronchiectasis was defined as either a change in one or more of the common symptoms of bronchiectasis (sputum volume or purulence, dyspnea, cough, and fatigue/malaise) or the onset of new symptoms (fever, pleurisy, or hemoptysis) (11).

#### Chronic dyspnea

The level of chronic dyspnea was assessed using the modified Medical Research Council (MMRC) dyspnea scale (15). Chronic dyspnea was graded from 0 to 4 in accordance with the description of breathlessness.

#### Radiographic extent of bronchiectasis

The radiographic extent of bronchiectasis was determined according to established computed tomography criteria using the following scoring system: grade 1: localized bronchiectasis affecting one or part of one bronchopulmonary segment, grade 2: bronchiectasis in more than one bronchopulmonary segment (extensive), and grade 3: generalized cystic bronchiectasis (16).

#### Chronic colonization by P. aeruginosa

Gram-stained sputum samples with >25 polymorphonuclear leukocytes and <10 squamous cells per field using a low-magnification lens were considered to be valid sputum samples. Valid sputum samples were obtained from all patients and processed for qualitative bacterial culture. Chronic colonization by *P. aeruginosa* was defined as at least three isolates of *P. aeruginosa* over a 3-month period and at least two isolates 3 months apart over a 1-year period (11).

#### Systemic inflammation

The peripheral blood C-reactive protein level was assessed by immunonephelometry (Cardio-Phase, Dade Behring Marburg GmbH, USA), and the erythrocyte sedimentation rate was measured using the Westergren method.

### Follow-up study

The maximum follow-up duration was 51 months. Follow-up examinations were performed at 3-month intervals. During each follow-up, we recorded severity of respiratory symptoms, frequency of acute exacerbations, number of hospital admissions due to non-CF bronchiectasis, and information regarding survival. For patients who could not be followed up, an effort was made to contact the patient by telephone to obtain information. Survival rates were determined at 1 to 4 years.

### Outcomes and prognosis

Two outcome parameters were prospectively recorded: the number of hospitalizations each year and the mortality rate. BMI was considered for its predictive value of outcomes, together with demographic data, pulmonary function, arterial blood gas analyses, C-reactive protein level, erythrocyte sedimentation rate, radiographic extent of bronchiectasis, and chronic colonization by *P. aeruginosa*.

### Statistical analysis

Descriptive data are reported as mean±SD or number (%). Analysis of variance was used to compare normally distributed variables among three or more groups; the Student-Newman-Keuls-*q* test was used for multiple comparisons. When data were not normally distributed, log transformation of the non-normal variables was performed before analysis of variance. Comparisons between qualitative variables were performed with the chi-squared test or Fisher’s exact test when necessary. Spearman rank correlation analysis was performed to analyze whether there was a correlation between two ordered categorical variables. Univariate and multivariate regression analyses were used to study the determinants of the risk of hospitalization. For survival analysis, parameters with a significant impact on survival in a univariate Cox model analysis were tested in a multivariate Cox proportional hazard model analysis. The survival process was described by Kaplan-Meier survival analysis. The log-rank test was used to test differences in the cumulative survival curves. Statistical analyses were performed using SPSS Statistics for Windows, Version 19.0 (SPSS Inc., USA). A P value of <0.05 was considered statistically significant.

## Results

### General characteristics

The baseline characteristics of the 339 patients with non-CF bronchiectasis are shown in Table 1. In total, 78.47% of the patients were lifetime nonsmokers. The mean BMI was 21.90 kg/m^2^ among all patients, and the prevalence of underweight patients was high (28.61%). The mean MMRC dyspnea score of 1.95 demonstrated a moderate severity of breathlessness. Among all 339 patients, 118 patients’ arterial blood gas analysis results met the diagnostic criteria for respiratory failure. Patients with normal ventilatory function only accounted for 29.20% of all patients. Obstructive ventilatory dysfunction was the most common pattern of pulmonary dysfunction (56.67%), followed by mixed ventilatory dysfunction (35.42%) and restrictive ventilatory dysfunction (7.91%). All patients underwent sputum sample collection and analysis. In total, 179 patients’ sputum specimens tested positive (52.80%). The most common isolated pathogen was *P. aeruginosa* (77.09%). Other pathogens were *Acinetobacter baumannii* (5.59%), *Haemophilus parainfluenzae* (4.47%), *Candida albicans* (4.47%), *Aspergillus* spp. (3.35%), *Klebsiella pneumoniae* (2.23%), *Escherichia coli* (2.23%) and *Staphylococcus haemolyticus* (0.57%). The underlying etiologies of non-CF bronchiectasis among the patients in this study are listed in Table 2. Underlying causes were identified in 135 patients (39.83%). However, no cause could be established in 204 patients (60.17%); these patients were considered to have idiopathic non-CF bronchiectasis.

**Table:**
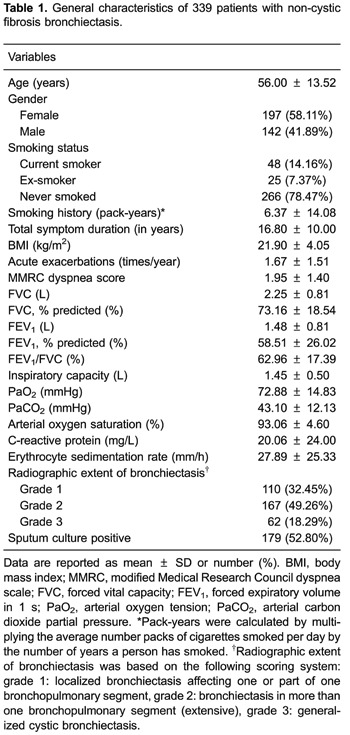


**Table:**
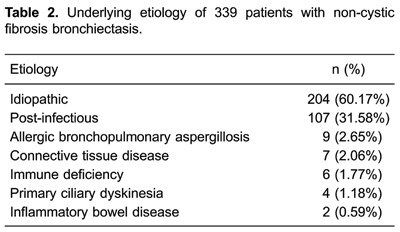


### Comparison among the four study groups

The number of patients in the 4 study groups were as follows: underweight group, 97 patients (28.61%); normal weight group, 173 patients (51.03%); overweight group, 55 patients (16.23%); and obese group, 14 patients (4.13%). Comparisons of the demographic data and clinical variables among the four groups are reported in Table 3. The following variables were significantly different among the four groups: total symptom duration in years, acute exacerbations, FVC, FEV_1_/FVC, inspiratory capacity, PaO_2_, C-reactive protein level, erythrocyte sedimentation rate, and chronic colonization by *P. aeruginosa*. In addition, acute exacerbations, the C-reactive protein level, and the erythrocyte sedimentation rate in the underweight group were significantly higher than those in the other three groups (P<0.05), while the inspiratory capacity in the underweight group was significantly lower than that in the other three groups (P<0.05). Total symptom duration in years and rate of chronic colonization by *P. aeruginosa* in the underweight group were significantly higher than those in the normal weight and overweight groups (P<0.05), while FVC, FEV_1_/FVC, and PaO_2_ were significantly lower than those in the normal weight and overweight groups (P<0.05). However, the differences in the symptom duration, FVC, FEV_1_/FVC, and PaO_2_ between the underweight and obese groups were not statistically significant. BMI was negatively correlated with the radiographic extent of bronchiectasis according to Spearman rank correlation analysis (*r*_s_=-0.312, P<0.001).

**Table:**
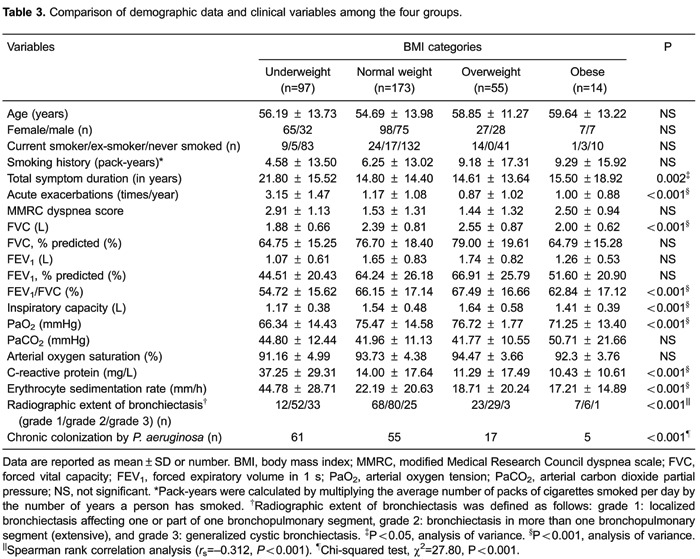


### Determinants of hospitalization risk

As shown in Table 4, the factors associated with a risk of hospitalization in the univariate regression analysis were age, total symptom duration in years, MMRC dyspnea score, BMI, FVC, predicted percentage of FVC, FEV_1_, predicted percentage of FEV_1_, FEV_1_/FVC, inspiratory capacity, PaO_2_, PaCO_2_, arterial oxygen saturation, C-reactive protein level, and erythrocyte sedimentation rate (P<0.05). Sex was not associated with the risk of hospitalization. Only the MMRC dyspnea score, BMI, erythrocyte sedimentation rate, C-reactive protein level, and total symptom duration in years appeared as independent predictors of hospitalization in the multivariate analysis. BMI was negatively correlated with the risk of hospitalization (standard regression coefficient=-0.26, P<0.001), while the MMRC dyspnea score, erythrocyte sedimentation rate, C-reactive protein level, and total symptom duration in years were positively correlated with the risk of hospitalization. In addition, the MMRC dyspnea score and BMI had more significant effects on the risk of hospitalization than did the erythrocyte sedimentation rate, C-reactive protein level, or total symptom duration.

**Table:**
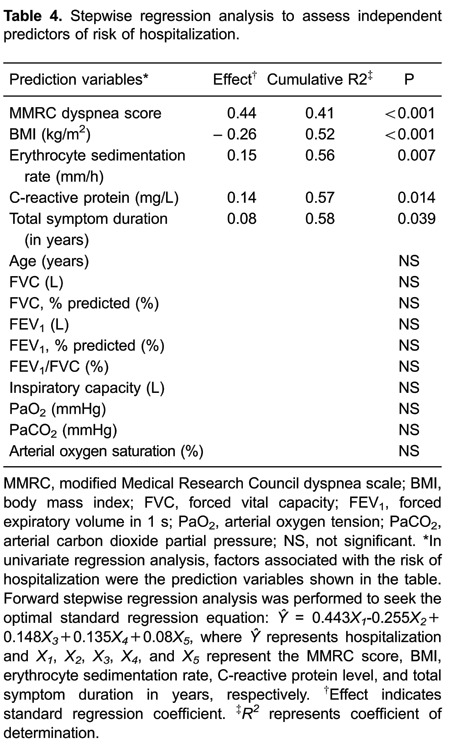


### Follow-up and survival

**Figure 1:**
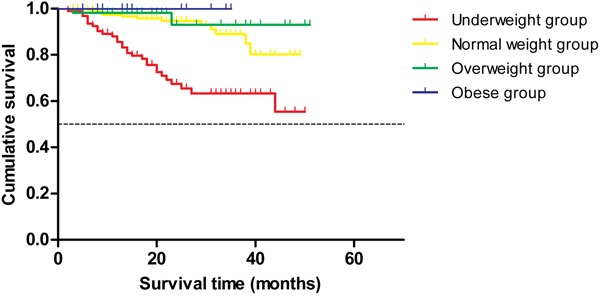
Differences in the cumulative survival curves among the four groups. The cumulative survival curves were statistically different among the four groups according to the log-rank test (χ^2^=31.67, P<0.001). Mortality gradually increased as BMI decreased according to a trend test (χ^2^=35.16, P<0.001).

The minimum and maximum follow-up times were 2 and 51 months, respectively. Survival was recorded during a follow-up of 21.70±12.38 months. Forty-three patients died, and all died of respiratory and circulatory failure. The 1-, 2-, 3-, and 4-year cumulative survival rates were 94%, 86%, 81%, and 73%, respectively. As shown in Figure 1, the cumulative survival curves were statistically different among the four groups (χ^2^=31.67, P<0.001), and the underweight group had the lowest cumulative survival rate. Moreover, the mortality rate increased gradually as the BMI decreased according to a trend test (χ^2^=35.16, P<0.001).

### Predictive factors of mortality

Predictive factors of mortality according to Cox proportional hazard model analysis are reported in Table 5. Parameters with a significant impact on survival after univariate Cox model analysis were PaCO_2_, inspiratory capacity, age, BMI, predicted percentage of FEV_1_, total symptom duration in years, predicted percentage of FVC, FEV_1_/FVC, PaO_2_, C-reactive protein level, erythrocyte sedimentation rate, radiographic extent of bronchiectasis, and chronic colonization by *P. aeruginosa*. However, when these parameters were tested in a multivariate Cox proportional hazard model, mortality was not influenced by the total symptom duration in years, predicted percentage of FVC, FEV_1_/FVC, PaO_2_, C-reactive protein level, erythrocyte sedimentation rate, radiographic extent of bronchiectasis, or chronic colonization by *P. aeruginosa*. Five parameters were independently associated with survival in the multivariate analysis: PaCO_2_, inspiratory capacity, age, BMI, and predicted percentage of FEV_1_. Low values for BMI, inspiratory capacity, and predicted percentage of FEV_1_ and high values for PaCO_2_ and age were significantly associated with increased mortality.

**Table:**
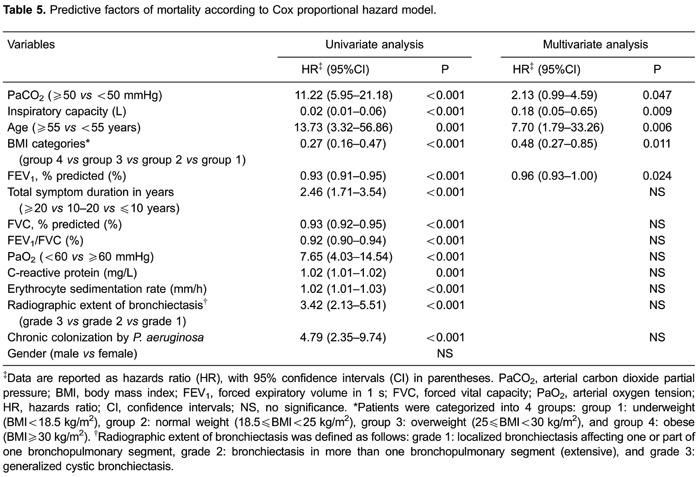


## Discussion

The main findings of the present study are as follows: an underweight status was highly prevalent among patients with non-CF bronchiectasis; BMI was associated with indicators reflecting disease severity, and patients with a lower BMI were prone to developing more acute exacerbations, worse pulmonary function, amplified systemic inflammation, and chronic colonization by *P. aeruginosa*; and BMI was a major determinant of hospitalization and death risks while a low BMI was associated with an unfavorable prognosis.

The prevalence of nutritional depletion in patients with non-CF bronchiectasis has not been fully studied. In the present study, we found a high prevalence of an underweight status among patients with non-CF bronchiectasis (28.61%). This percentage is similar to those in other studies that reported nutritional depletion rates of 14% and 30% as analyzed by BMI (6,7). Therefore, weight loss was a frequently occurring phenomenon in patients with non-CF bronchiectasis.

The association between BMI and pulmonary function in patients with chronic respiratory diseases has been recognized for many years, but it has mainly been documented in patients with COPD. In one study of patients with COPD, BMI was positively associated with FEV_1_/FVC and the predicted percentage of FEV_1_ (17). In another study, the incidence of airflow obstruction (defined as an FEV_1_/FVC of <70%) in patients with COPD was significantly higher in those with a BMI of <18.5 kg/m^2^ than in those with a BMI of ≥18.5 kg/m^2^ (18). Several recent studies have documented a clear association between a low BMI and poor pulmonary function in patients with bronchiectasis. A retrospective analysis of patients with bronchiectasis reported that BMI was positively correlated with FEV_1_ (7). In a multicenter cross-sectional survey of outpatients on long-term oxygen therapy or home mechanical ventilation (including 39 patients with bronchiectasis), BMI was found to be positively associated with the predicted percentage of FVC and predicted percentage of FEV_1_ (19). Additionally, a low BMI was significantly associated with a low inspiratory capacity (20). The results of our study are in line with those of previous studies. We demonstrated that BMI was positively correlated with FVC, FEV_1_/FVC, and inspiratory capacity. Our study has shown that patients with non-CF bronchiectasis with a lower BMI were prone to developing worse pulmonary function. The hypothesis was that weight loss (particularly loss of muscle mass) caused by malnutrition might promote a decrease in respiratory muscle strength, eventually leading to worse pulmonary function (21). Long-term longitudinal analyses are needed to better identify the effect of a low BMI on the reduction of pulmonary function.

Both the C-reactive protein level and erythrocyte sedimentation rate have known value as markers of systemic inflammation and are indirect markers of disease activity and quality of life for patients with non-CF bronchiectasis (11). The association between systemic inflammation and nutritional depletion in patients with chronic respiratory diseases has recently become an area of increasing research interest. In one study, the authors reported that an overflow of inflammatory cytokines might lead to malnutrition in patients with COPD (22). Moreover, in a cross-sectional study of patients with COPD, C-reactive protein levels were found to be significantly higher in patients with a low BMI (≤21 kg/m^2^) than in those with a normal-to-high BMI (>21 kg/m^2^), and an elevated C-reactive protein level was considered to be an indicator of malnutrition in patients with COPD (23). Consistent with other studies of chronic respiratory diseases, we found that BMI was negatively correlated with the C-reactive protein level and erythrocyte sedimentation rate in patients with non-CF bronchiectasis and that underweight patients had significantly higher C-reactive protein levels and erythrocyte sedimentation rates than did other patients. Our data suggest a link between a low BMI and increased systemic inflammation. Data from the current study support a previously proposed concept of disease-related malnutrition, in which disease is a major factor for malnutrition and the risk of malnutrition increases with disease severity (24). The mechanism of disease-related malnutrition is multifactorial, but the combination of decreased nutritional intake, increased energy and protein requirements, and the presence of inflammation probably plays the central role (25). A recently proposed hypothesis states that inflammation plays a key role in the pathogenesis of disease-related malnutrition (26). Our findings do not allow for the establishment of a causal relationship between inflammation and weight loss in patients with non-CF bronchiectasis, but may provide insights into the cause of weight loss and malnutrition in these patients.

Lower respiratory tract infections repeatedly occur in patients with non-CF bronchiectasis. In one study, sputum cultures tested positive for *K. pneumoniae* or *Streptococcus pneumoniae* during the initial or stable phase of bronchiectasis. With disease progression, however, *P. aeruginosa* replaced other pathogens and colonized the sputum (27). Very few studies have investigated the relationship between weight loss and chronic colonization by *P. aeruginosa.* One study of patients with COPD found that a low BMI was one of the independent determinants of a positive sputum culture and that the most common pathogen isolated was *P. aeruginosa* (28). The present study showed that BMI was negatively correlated with the rate of chronic colonization by *P. aeruginosa*, demonstrating that patients with non-CF bronchiectasis with a low BMI are more susceptible to chronic colonization by *P. aeruginosa*. Published data suggest that malnutrition is an independent factor associated with nosocomial infections (29). The causal relationship between weight loss and *P. aeruginosa* infection has recently been explored. Mouse models of chronic bronchopulmonary infection with *P. aeruginosa* exhibited significant weight loss, and the weight loss was directly correlated with the degree of pulmonary inflammation (30). Using a mouse model of *P. aeruginosa* infection, Kishta et al. (31) showed that nutritionally derived products with anti-inflammatory and antioxidant properties limited the bacterial burden and protein oxidation in *P. aeruginosa* lung infection. The hypothesis was that *P. aeruginosa* lung infection is associated with a marked inflammatory response and oxidative stress and that there is an intimate relationship among *P. aeruginosa* lung infection, inflammation, and weight loss.

One of the main concerns in patients with non-CF bronchiectasis is identification of the determinants of hospitalization because hospitalization is associated with a high mortality rate and is the main source of costs. In the present study, the number of hospitalizations was independently determined by a high MMRC dyspnea score, low BMI, elevated erythrocyte sedimentation rate, elevated C-reactive protein level, and rising total symptom duration in years. Dyspnea is one of the main symptoms of bronchiectasis, and Onen et al. (10) reported that dyspnea as measured by the MMRC dyspnea score was correlated with prognosis in patients with non-CF bronchiectasis. Additionally, a prospective study showed that a low BMI was associated with an increased risk of hospitalization in patients with non-CF bronchiectasis (7). Consistent with these results, our findings also demonstrated the relationship between a low BMI and risk of hospitalization. The hypothesized mechanism was the vicious circle of malnutrition and infection. A possible intermediate pathway could be immunodeficiency secondary to malnutrition. It has been proposed that malnutrition might influence the organism’s defense processes by impairing the lymphohematopoietic organs and modifying the immune response (32). Therefore, malnourished individuals have a greater susceptibility to infection. In turn, repeated infections and frequent exacerbations lead to weight loss by reduced dietary intake and increased resting energy expenditure (33).

The influence of a high PaCO_2_ on survival was clearly demonstrated in a 4-year follow-up study of patients with bronchiectasis (10). The level of hypercapnia reflects the severity of the respiratory impairment. For this reason, patients with chronic hypercapnia during follow-up have a worse prognosis than do patients with normocapnia (10). The predicted percentage of FEV_1_ also has significant predictive power with respect to mortality, which confirms the recent hypothesis proposed by Martinez-Garcia et al. (4). An observational prospective study of hospitalized patients in Brazil showed that malnourished patients had a higher risk of death than did well-nourished patients (34). Furthermore, a prospective cohort study of patients with bronchiectasis showed that a low BMI was an independent predictor of long-term mortality (10). The main finding of our study is that nutritional depletion, as evaluated by BMI, not only correlated with the risk of hospitalization, but also appeared to be an independent predictor of mortality in patients with non-CF bronchiectasis. We found that, among patients with non-CF bronchiectasis, those with a low BMI had a worse prognosis than those with a normal-to-high BMI. The suggested mechanism for the higher mortality in patients with a lower BMI may be an impaired immune response with respiratory muscle weakness. Previous studies have found that maintaining an optimal nutritional status and achieving protein balance during routine care was important in the prevention of muscle loss and further improvements in the clinical and overall outcomes of patients with CF (35). Likewise, the Cochrane database of systemic reviews of randomized controlled trials suggested that nutritional support improves the prognosis of patients with COPD and is useful for their comprehensive care (36). Nutritional support as an additional therapy for non-CF bronchiectasis has often been neglected. Randomized controlled clinical trials are needed to explore the impact of nutritional management on clinical outcomes of patients with non-CF bronchiectasis.

The present study has limitations that must be acknowledged. First, although BMI is used to screen patients for malnutrition, it may not accurately reflect the nutritional status. Many tests are used to assess the nutritional status, including objective methods (such as anthropometry and laboratory tests) and subjective methods (such as subjective global assessment and the Nutrition Risk Screening 2002). Subjective global assessment is a good nutritional assessment tool and has been validated for the prediction of poor clinical outcomes in hospitalized patients (37). Further studies should use more comprehensive methods to evaluate the nutritional status of patients with non-CF bronchiectasis and to explore the mechanism of the association between malnutrition and prognosis. Second, we did not have a measure of body composition, such as fat-free mass. Published data show that loss of skeletal muscle mass is the main cause of weight loss in patients with COPD and that the fat-free mass index provides information in addition to the BMI (38). Further studies are needed to address this. Third, high-resolution computed tomography scans of the chest in patients with bronchiectasis do not exclusively show dilated bronchi; they also show signs of small airway involvement, such as air trapping, the tree-in-bud pattern, bronchiolectasis, and excess mucus (11). Imaging signs of small airway involvement are reportedly an important modality with which to monitor the progression of bronchiectasis due to CF (39). Unfortunately, we did not evaluate the imaging signs of small airway involvement. Further studies will be conducted to evaluate the radiographic extent of bronchiectasis in patients with non-CF bronchiectasis in detail. Fourth, some examinations were only performed at baseline. We failed to record the changes in pulmonary function, arterial blood gas analyses, or high-resolution computed tomography scans of the chest from baseline into the follow-up period. Long-term longitudinal analyses are needed to demonstrate the relationship between the BMI and rate of lung function decline.

In the present study, an underweight status was highly prevalent among patients with non-CF bronchiectasis. Patients with a lower BMI were prone to increased acute exacerbations, worse pulmonary function, amplified systemic inflammation, and chronic colonization by *P. aeruginosa*. BMI was one of the major determinants of hospitalization and death risks in patients with non-CF bronchiectasis. BMI should be considered in the routine assessment of patients with non-CF bronchiectasis.
